# Field evaluation of a new particle concentrator- electrostatic precipitator system for measuring chemical and toxicological properties of particulate matter

**DOI:** 10.1186/1743-8977-5-15

**Published:** 2008-11-11

**Authors:** Zhi Ning, Markus Sillanpää, Payam Pakbin, Constantinos Sioutas

**Affiliations:** 1 University of Southern California, Department of Civil and Environmental Engineering, 3620 South Vermont Avenue, Los Angeles, CA 90089, USA; 2 Finnish Institute of Occupational Health, Topeliuksenkatu 41 a A, FI-00250, Helsinki, Finland

## Abstract

**Background:**

A newly designed electrostatic precipitator (ESP) in tandem with Versatile Aerosol Concentration Enrichment System (VACES) was developed by the University of Southern California to collect ambient aerosols on substrates appropriate for chemical and toxicological analysis. The laboratory evaluation of this sampler is described in a previous paper. The main objective of this study was to evaluate the performance of the new VACES-ESP system in the field by comparing the chemical characteristics of the PM collected in the ESP to those of reference samplers operating in parallel.

**Results:**

The field campaign was carried out in the period from August, 2007 to March, 2008 in a typical urban environment near downtown Los Angeles. Each sampling set was restricted to 2–3 hours to minimize possible sampling artifacts in the ESP. The results showed that particle penetration increases and ozone concentration decreases with increasing sampling flow rate, with highest particle penetration observed between 100 nm and 300 nm. A reference filter sampler was deployed in parallel to the ESP to collect concentration-enriched aerosols, and a MOUDI sampler was used to collect ambient aerosols. Chemical analysis results showed very good agreement between the ESP and MOUDI samplers in the concentrations of trace elements and inorganic ions. The overall organic compound content of PM collected by the ESP, including polycyclic aromatic hydrocarbons (PAHs), hopanes, steranes, and alkanes, was in good agreement with that of the reference sampler, with an average ESP -to -reference concentration ratio of 1.07 (± 0.38). While majority of organic compound ratios were close to 1, some of the semi-volatile organic species had slightly deviated ratios from 1, indicating the possibility of some sampling artifacts in the ESP due to reactions of PM with ozone and radicals generated from corona discharge, although positive and negative sampling artifacts in the reference filter sampler cannot be ruled out.

**Conclusion:**

The very good overall agreement between ESP and reference samplers makes it an attractive alternative to filters and biosamplers for chemical and toxicological evaluation of PM properties, including the possibility of conducting direct in vitro cell exposures. Moreover, the concentration enrichment of ambient aerosols by the VACES allows for short-term exposure studies, which preserve cell viability and enable studies to PM generated from specific sources and-or formation mechanisms in the atmosphere.

## Background

There is extensive epidemiological evidence associating ambient particulate pollution with adverse health effects in humans [1,2]. Nevertheless, fundamental uncertainty and disagreement persist regarding the physical and chemical properties of particles (or unidentified confounding environmental influences) that influence health risks, the pathophysiological mechanisms that are operative, and what air quality regulations should be adopted to deal with the health risks attributed to particulate matter (PM) [3,4].

Continuing advancements in techniques to improve the chemical and physical characterization of aerosol particles provide insights into aerosol formation and atmospheric evolution and improve our understanding on their environmental and health impacts. Important atmospheric parameters influencing the ambient PM concentrations and their characteristics, such as temperature, relative humidity, wind direction and speed, and mixing height, fluctuate in time scales that are on the order of few hours or shorter. In an ideal system, PM collection for measurement of their chemical and toxicological characteristics should be done using direct and on line methods. The Versatile Aerosol Concentration Enrichment System (VACES) has been used for in-vivo human or animal exposure studies for years. The same system has been used in tandem with a liquid particle collector (Biosampler™, SKC Inc, Eighty-four, PA, USA) to collect in-vitro samples into aqueous suspensions for indirect cell exposure. Nevertheless, this novel system cannot provide PM for direct cell exposures

In vitro assays, which are used for measuring the toxicological properties of PM, generally require high quantities (order of several mg of PM) for chemical and biological analyses, therefore high volume filter samplers are used. PM is typically collected on substrates or filters such as quartz and Teflon, and is subsequently extracted by means of a solvent. If ultra pure water is used for extraction, insoluble PM species, which may be toxicologically important, will likely be extracted with low efficiency. If an organic solvent is used for extraction, the solvent should be removed prior to the in vitro bioassay, given that the solvent itself may be toxic to cell cultures or elicit significant biological responses. This is normally done by means of lyophilization, which removes the solvent by either applying a vacuum, or by purging the suspension with a stream of an inert gas such as N_2_. This process will undoubtedly remove potentially toxic PM-bound labile species, such as semi-volatile organics. The sonication process itself may introduce sampling biases, including incomplete particle removal, physical changes (agglomeration, possibly de-aggregation) as well as altering the chemical or biological properties of PM. An extensive literature discusses sampling artifacts associated with the use of filters as PM collectors [5,6]. These include loss of labile species, such as ammonium nitrate and more importantly organics from PM on the filter during prolonged sampling periods; (for quartz filters) adsorption of vapor phase organics; (for all filters) reactions between particle and incoming gases, for example reduction and transformation of PAH with O_3 _to oxy-PAH [7].

Several approaches have been developed as an alternative to filtration, including collecting particles in a fluid using a combination of particle concentration, followed by impaction and centrifugation [8,9]. These particle concentrators are portable and have been shown to increase ambient particle levels by a factor of approximately 20 without significantly affecting particle properties such as size [10], bulk chemistry [8,11] or single particle chemistry [12] and morphology [8]. These concentrators can be used to provide elevated ambient PM exposures to animal or human subjects, as well as to collect a large amount of PM material in aqueous solution suitable for subsequent toxicological assays. The main advantage of these technologies over filtration is that PM collection resembles a system closer to real world exposure and deposition onto human cells in respiratory system. Detailed studies cited above have revealed few and generally negligible artifacts during PM collection. Moreover, the concentration enrichment process minimizes volatilization losses in conventional particle collectors that could be applied downstream of the concentrator, such as impactors and filters, from ~50–70% to less than 10%, as demonstrated by Chang et al. [13]. Disadvantages of these technologies include a complicated operation, requiring highly skilled and properly trained personnel.

To overcome some of the aforementioned shortcomings of conventional particle collection methodologies, we developed a new sampling system, the in-vitro electrostatic collector, to collect ambient particles for either in vivo or in-vitro toxicity studies [14]. The system consists of two units: first, particles are concentrated by means of the Versatile Aerosol Concentration Enrichment System (VACES), and are subsequently drawn through an electrostatic precipitator (ESP). The particle sample can be collected on a petri dish that contains cell cultures, or on any other desirable substrate suitable for particle collection and analysis. These substrates are placed on top of the grounded electrode of the ESP. The enriched aerosol concentration after the VACES make it possible to sample for short time intervals, which favors cell viability and exposure characterization, as shown by Sillanpää et al. [14]. The laboratory tests showed that collection efficiency under optimized conditions is higher than 95% across all particle diameters measured (18 nm to 3.0 μm), regardless of aerosol type.

This paper is an expansion of our previous work on the VACES-ESP collector described by Sillanpää et al. [14]. That study was conducted using mostly laboratory particles. Limited data were collected using ambient indoor and outdoor aerosols, although what was observed produced results consistent with those in the laboratory. In this paper, we expand our previous work to validate the performance of VACES-ESP using ambient aerosols collected in an urban environment in downtown Los Angeles, CA. Of particular interest in the present study was the ability of the VACES-ESP to collect effectively species such as trace elements and inorganic ions, as well as organic compounds, such as polycyclic aromatic hydrocarbons (PAH), hopanes and steranes, and alkanes, all of which have been hypothesized to lead to health effects attributable to PM [15,16].

## Results and discussion

### Overview of sampling campaign results

The particle parameters measured during the sampling period have been summarized in Table 1. The penetration of concentration-enriched particles through the ESP, based on the particle number measurements, varied in the range of 2.5–10% during the sampling campaign. This is in good agreement with the ESP penetration experiments done with different types of laboratory test particles [14]

**Table 1:** Particle number and mass concentrations measured during sampling and sample distribution for chemical analysis

	Particle number		PM_2.5 _mass concentration			
			
Test No.	Ambient concentration (particles/cm^3^)	ESP Penetration (%)^a^	Ambient concentration (μg/m^3^)	Enriched concentration (μg/m^3^)	Enrichment Factor (EF)^b^	Chemical analysis
1	16600 ± 2900	3.5	23.2	370.8		
2	21600 ± 5500	3.6	26.3	665.5	25.3	ICP-MS
3	14600 ± 3000	4.7	46.5	940.3	20.2	GC-MS
4	10400 ± 2900	5.0	29.5	827.1	28.0	ICP-MS
5	15900 ± 5300	3.5	11.9	252.8	21.2	GC-MS
6	21600 ± 9200	2.6	29.3	310.0	10.6	ICP-MS
7	12200 ± 4800	3.8	18.9	453.0	24.0	GC-MS
8	30800 ± 6200	3.1	19.0	323.0	17.0	IC
9	14000 ± 3000	9.6	17.0	463.2	27.2	GC-MS
10	15000 ± 4000	7.7	16.5	490.6	29.7	GC-MS
11	15000 ± 5000	9.6	17.7	265.9	15.0	GC-MS
12	14000 ± 3000	7.4	20.7	228.6	11.0	GC-MS
13	13000 ± 3000	8.9	14.5	252.5	17.4	GC-MS
14	12000 ± 3000	8.7	16.5	289.8	17.6	GC-MS

^a ^ESP penetration was determined as the ratio of particle number concentration penetrating ESP to the enriched particle number concentration before ESP.

^b ^EF (Enrichment factor) was determined as the ratio of enriched PM_2.5 _mass concentration from reference samplers to ambient PM_2.5 _mass concentrations from MOUDI.

The ambient PM_2.5 _mass concentration ranged from 11.9 μg/m^3 ^to 46.5 μg/m^3^, while the enriched concentration varied in the range of 253 – 940 μg/m^3^. The average enrichment factor (EF) over the entire sampling campaign was 20.1 ± 6.1, which is very close to the ideal value of 22 [9]. Average number- based concentration enrichment factor during the sampling campaign was 15.3 ± 3.7. The measured uncertainties during the sampling period were due to the variation of ambient conditions. Nevertheless, the comparison between the concentration-enriched reference samples and the MOUDI was in practically excellent overall agreement, thus corroborating the use of this filter sampler as a reference to which the ESP concentrations could be compared.

The distribution of samples for different chemical analysis was based on the obtained particle mass loadings on various filters and substrates. Detailed description of the analytical methods used for different samples are listed in Table 1. Based on the overall mass loadings, samples #1 and #8 were selected for ion analysis, whereas trace elements analysis was done for samples #2, #4 and #6 as a composite sample. Since the concentrations of individual organic compounds are generally low in the urban atmosphere, the remaining filter samples were composited separately into four sets to acquire enough analytical mass for organic speciation by Gas Chromatography–Mass Spectrometry (GC-MS) method (The composite sets include sample #3; samples #5 and #7; sample #9, #11 and #13; sample #10, #12 and #14). The ionic, elemental, and organic compositions of these samples are described in the following sections.

In addition, separate tests were conducted with fluorescent particles to determine the uniformity of the particle deposition on the ESP using the method presented by Sioutas et al. [17]. The tests showed ± 25% differences from the mean of collected mass along the substrate, with higher particle deposition density closer to the entrance of the ESP. This deposition pattern can become more uniform by simply changing periodically (e.g., every 15 minutes) the air flow direction inside the ESP using a switch valve. These results will be described in a separate manuscript in greater detail [18].

### Particle penetration through ESP at different flow rates

**Figure 1:**
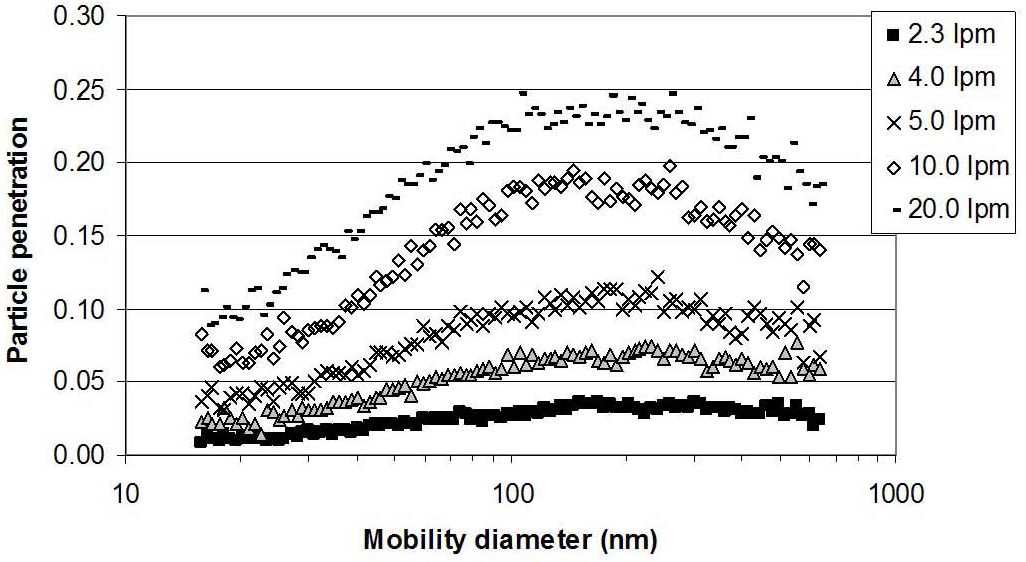
Penetration of 16 to 640 nm (NH_4_)_2_SO_4 _particles at different ESP flow rates (5.3 kV/cm).

Particle penetration is defined as the ratio of particle concentration measured after the ESP to that entering the ESP. The original laboratory validation of the ESP was based on the measurements done at a constant sampling flow rate, i.e. 1.8 l min^-1 ^[14]. Since the ESP may be used in numerous applications, which may require different sampling flow rates, its collection efficiency was tested with laboratory ammonium sulfate aerosols at five different sampling flow rates. The particle collection efficiency at all size ranges decreases with increasing flow rate, as shown in Figure 1. At all flow rates the lowest ESP collection efficiency was observed for particles between 100 nm and 300 nm in mobility diameter. Similar results were also observed in many experimental studies as reported in literature [19-21]. The electrostatic velocity of particles in any ESP is a combination of the number of charges acquired by the particles and the particle mobility. Although particle-charging efficiency decreases with particle size, the mobility increases rapidly with the decreasing particle size. Consequently, there exists a theoretical minimum collection efficiency in the size range of 0.1 to 0.5 μm [22]. In the present study, the penetration ranged from 6–7% at 4 l min^-1 ^to 22–24% at 20 l min^-1 ^in the size range of 100 nm to 300 nm. This suggests that relatively high collection efficiencies can still be reached at sampling flow rates as high as 20 l min^-1^.

**Figure 2:**
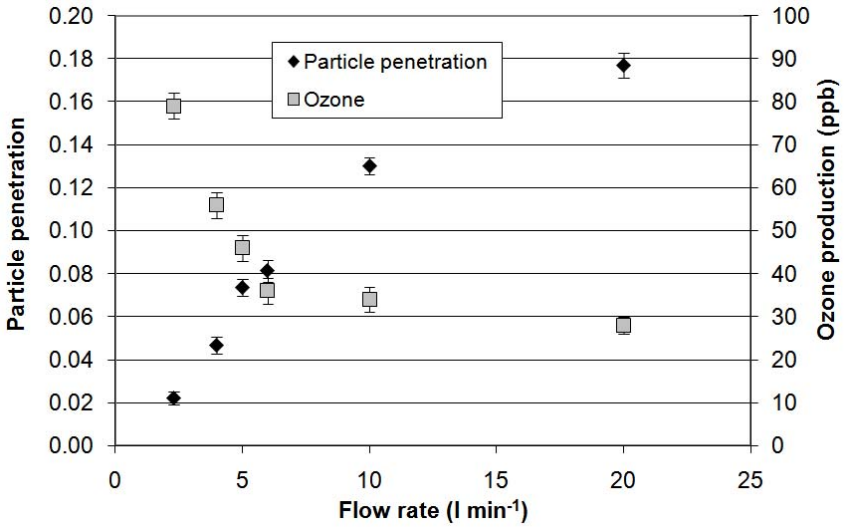
(NH_4_)_2_SO_4 _particle number and ozone concentrations as a function of ESP flow rate (5.3 kV/cm).

In the second set of laboratory experiments, ozone produced by the ESP corona discharge was investigated at flow rates ranging from 2 to 20 l min^-1^, which correspond to residence times of 1.1–11 seconds, based on the geometric dimensions of the ESP. Figure 2 shows the penetration of ammonium sulfate particles and ozone production as a function of sampling flow rate. As expected, particle penetration increases and ozone concentration decreases with increasing sampling flow rate. Also, there is no significant change in ozone production at flow rates between 6 l min^-1 ^and 20 l min^-1^. During the field evaluation campaign, a flow rate of 4 l min^-1 ^was selected to achieve both low particle penetration and low ozone generation in an effort to minimize possible chemical artifacts in the ESP.

### ESP performance based on PM chemical components

**Figure 3:**
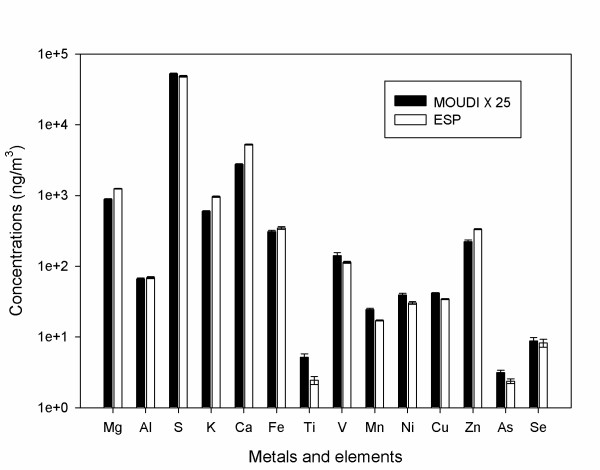
**Comparison of the concentrations of metals and elements from ESP and MOUDI samples**. Error bars represent the analysis uncertainties of the samples.

Fourteen individual metals and elements were analyzed using ICP-MS for ESP and MOUDI samples. Figure 3 shows the comparison of the concentrations of trace metal and element concentrations obtained by the ESP and MOUDI samplers. Data are shown in logarithmic scale to cover the wide range of measured concentrations. The average of the ratios of ESP and MOUDI ambient samples is 24.7 ± 5.1. Considering the wide range of ambient concentrations, spanning over 4 orders of magnitude, and the intrinsic uncertainties associated with the low ambient levels of some of these elements (particularly for the MOUDI data), the achieved enrichment factor is in very good agreement with the theoretical enrichment factor of 22. It should also be noted that the concentration enrichment determined by the inductively coupled plasma-mass spectroscopy (ICP-MS) analysis is also very close to the gravimetrically determined concentration enrichment (21.3 ± 8.2) for samples #2, #4 and #6 that were composited for this analysis, as shown in Table 1.

**Figure 4:**
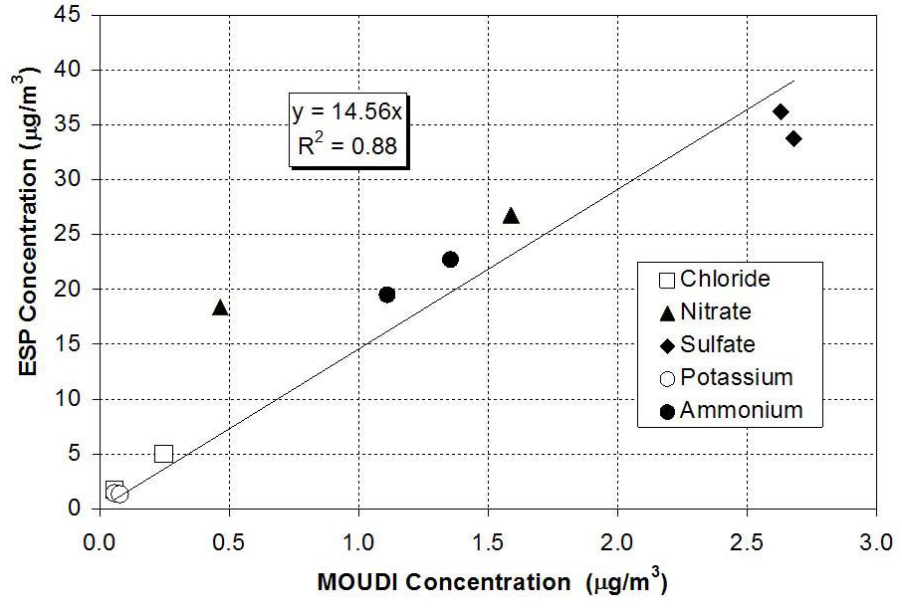
ESP versus MOUDI inorganic ion concentrations with linear regression.

Five inorganic ions were analyzed using ion chromatography (IC) for samples #1 and #8, respectively. Figure 4 shows the correlation plot between the ion concentrations from ESP and the MOUDI measurements. Linear regression showed a very good agreement between the two samplers, with a slope of 14.6 and a regression coefficient (*R*^2^) value of 0.88. There is one possible outlier for the nitrate measurements, indicating somewhat higher concentration enrichment than the overall mean. This could be due to either the unusually low (for Los Angeles) ambient nitrate levels of that experiment (i.e., 0.5 μg/m^3^), which may result in some uncertainty in the reported MOUDI values, or to potential negative artifacts due to loss of labile nitrate from the MOUDI filter [23]. The average concentration enrichment factor for these sets of samples based on gravimetric measurements was 16.50 (16.0 for sample #1 and 17.0 for sample #8), which was very close to the ideal EF of 18 obtained with an on-line SMPS during this set of experiments. The agreement in the enrichment factors based on PM mass and individual ions provides further corroboration of the integrity of the ESP in charging and collecting ionic PM species. The similarities in the ESP performance in collecting particle-bound trace elements and ions provide further evidence that the ESP's charging efficiency is not dependent on PM chemical components. Similar results were also observed in the previous laboratory test using artificial aerosols [14].

### ESP performance based on organic species

Compared to filter-based sampling methods, the ESP is less susceptible to adsorptive and evaporative artifacts, since the particle collection surface area in the ESP is significantly smaller than the effective surface area of a filter [24]. However, ESP samples are subject to alterations in particle chemical composition for two possible reasons. First, ozone generated during corona discharge in the ESP may alter the PM chemical composition by means of chemical reactions with the collected particles [25]; furthermore, free radicals and ions (i.e., O_2_^+^, O^+^, N_2_^+^, N^+^, NO^+ ^and H_3_O^+^) generated by corona discharge may react with both particles and vapors in the plasma region in the ESP [26]. Volckens and Leith [24] demonstrated that these corona discharge-related reaction artifacts can be alleviated by shortening the sampling time. To that end, each sampling period in the present study was restricted to 2–3 hours to minimize potential chemical artifacts in ESP, as we discussed earlier.

**Figure 5:**
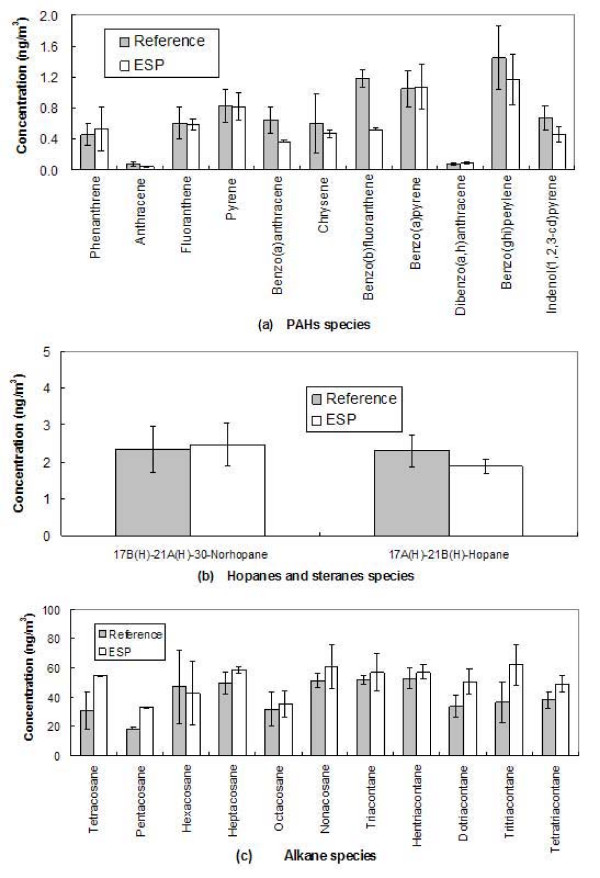
**ESP versus reference filter sampler for (a) PAHs, (b) hopanes and steranes, (c) alkanes concentrations**. Error bars represent the standard deviations of the analyzed samples.

Figure 5a shows the comparison of PM bound PAH concentrations from ESP and reference samples. Error bars represent the standard deviation of the analyzed samples. The ratios of ESP and reference samples for most of the quantified PAHs species are close to 1, except for benzo(a)anthracene (0.56), benzo(b)fluoranthene (0.44), and anthracene (0.57). The average ratio for all PAHs is slightly less than 1 (0.83 ± 0.06), suggesting that there might be some degradation of these semi-volatile organic species in ESP due to the aforementioned artifacts. However, the overall agreement between ESP and reference samplers (i.e., within 17%) is quite promising. Figure 5b and 5c show the comparison of the ESP and reference sampler concentrations for hopanes, steranes and alkanes. The average ESP to reference ratio for hopanes and steranes is 0.96 ± 0.15, and the ratio for alkanes is 1.29 ± 0.31. Overall, the agreement between the ESP and filter sampling methods for these generally non-labile and stable species should be considered very good and well within the experimental and analytical uncertainties.

**Figure 6:**
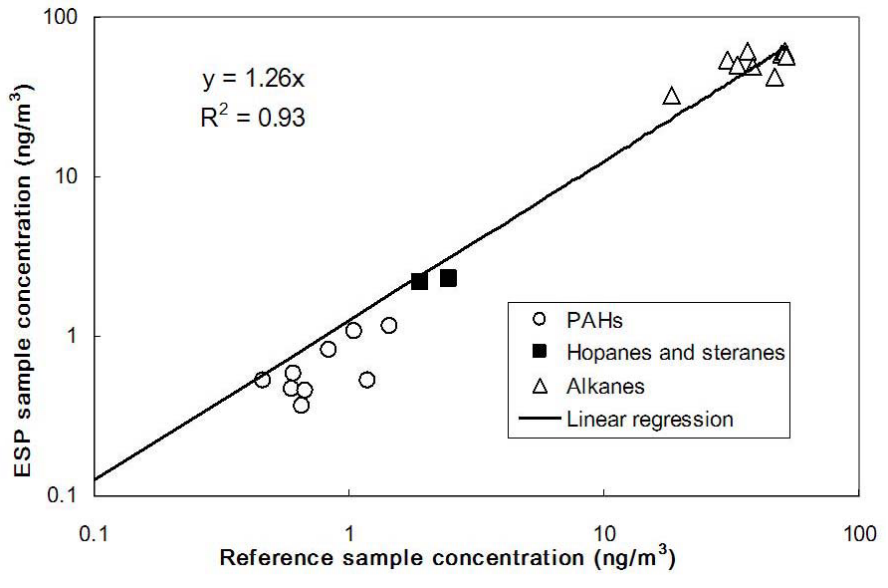
Correlation of the PAHs, hopanes and steranes, and alkanes concentrations from reference and ESP samples.

Figure 6 shows a plot of the linear regression between ESP and reference samples for PAHs, hopanes and steranes, and alkanes, all combined in one graph. The overall good agreement between these two sampling methods is evident. Linear regression between the ESP and reference filter concentrations showed a slope of 1.26 and regression coefficient of 0.93, while the average ESP-to-reference filter concentration ratio of individual organic species was 1.07 ± 0.38.

It should be noted that in our experiments, filter sampling has been used as the reference method to which the ESP concentrations could be compared. Our choice was based on the fact that filters to this day represent the most common methodology for PM sampling and analysis of any kind, whether gravimetric, chemical or toxicological. This by no means implies that the data obtained from the reference filter sampler (and for that matter the ESP) are artifact-free, especially for some organic compounds as well as nitrate, as we discuss in the introductory part of this paper. The interpretation of our results and the degree of agreement between the methods tested in this paper will thus need to be viewed with caution and treated with the appropriate caveats introduced by the lack of an ideal method for PM collection and analysis. Indeed, the existence of such a method would have made this study quite unnecessary. Nonetheless, the very good overall agreement between the ESP and reference filter methods certainly encourages the consideration of the ESP method as an attractive alternative to filters and biosamplers for chemical and toxicological PM analyses, including direct in vitro cell exposures.

## Conclusion

A newly designed Electrostatic Precipitator (ESP) in tandem with a Versatile Aerosol Concentration Enrichment System (VACES) was tested in an urban environment to validate its performance for collecting ambient particles for potential applications in the field of toxicology, including direct in-vitro cell exposure. To investigate the performance of the ESP, a reference sampler was deployed in parallel to collect concentration-enriched aerosols, and a MOUDI sampler was used to collect ambient aerosols. For each experimental set, the sampling time was restricted to 2–3 hours to minimize potential chemical artifacts related to the generated ozone and radicals during corona discharge. Also, previous cell exposure experiments with the ESP have shown that cell viability can be preserved after 2 hours of exposure to particle – free air [12].

Collected filter samples were analyzed to determine gravimetric mass concentrations as well as concentrations of various PM inorganic and organic chemical species from the ESP, reference and MOUDI samplers. Very good agreement between samplers was observed in the enrichment factors based on gravimetric PM mass and individual inorganic chemical species. Selected ESP and reference samples were analyzed for polycyclic aromatic hydrocarbons (PAH), hopanes, steranes, and alkanes. The majority of the PAH concentrations from ESP and reference samplers had a ratio close to 1, with few exceptions. The average ESP- filter ratio was 0.83 across all PAH species. While some degradation of certain PAH is possible in the EPS over prolonged sampling time, our comparisons are also confounded by the fact that semi-volatile organic species, such as PAHs, tend to induce both positive (by adsorption) and negative (by evaporation) artifacts in filter sampling. For stable, non-labile organic species, such as hopanes and steranes, and alkanes, the average ESP- filter ratios are relatively higher compared with PAHs (0.96 for hopanes and steranes, 1.29 for alkanes). The good overall agreement for organic species, with ESP to reference concentration ratio of 1.07, suggests that the short sampling times and generally low O_3 _generation in the present study tend to minimize the chemical sampling artifacts in the ESP.

The unique design of VACES-ESP system can effectively provide highly concentrated aerosols to a collection substrate that is suitable for any chemical or biological analysis, including collection onto a cell culture layer for conducting direct cell exposures to PM. The system allows for greatly shortened sampling durations, and delivers sufficient PM mass loadings to cell cultures in time periods that ensure cell viability. Our laboratory and field experiments have shown that the VACES-ESP system is a viable and promising technique that can be used as an alternative to conventional filtration and impaction methods for measuring chemical and toxicological properties of PM.

## Methods

### Ambient sampling site

Field validation measurements were carried out between August, 2007 and March, 2008 at an urban sampling site – the Particle Instrumentation Unit of the Southern California Particle Center and Supersite – near the University Park campus of the University of Southern California in Los Angeles. The sampling site is situated within 100–150 m of a major freeway and adjacent to a multi-story parking structure, representing a typical urban environment with mixed particle sources [27]. The sampling inlets were located at the roof of the trailer and within ca. 2 m of each other. All instruments were equipped with PM_2.5 _size selective inlets.

### Sampling setup and methodology

**Figure 7:**
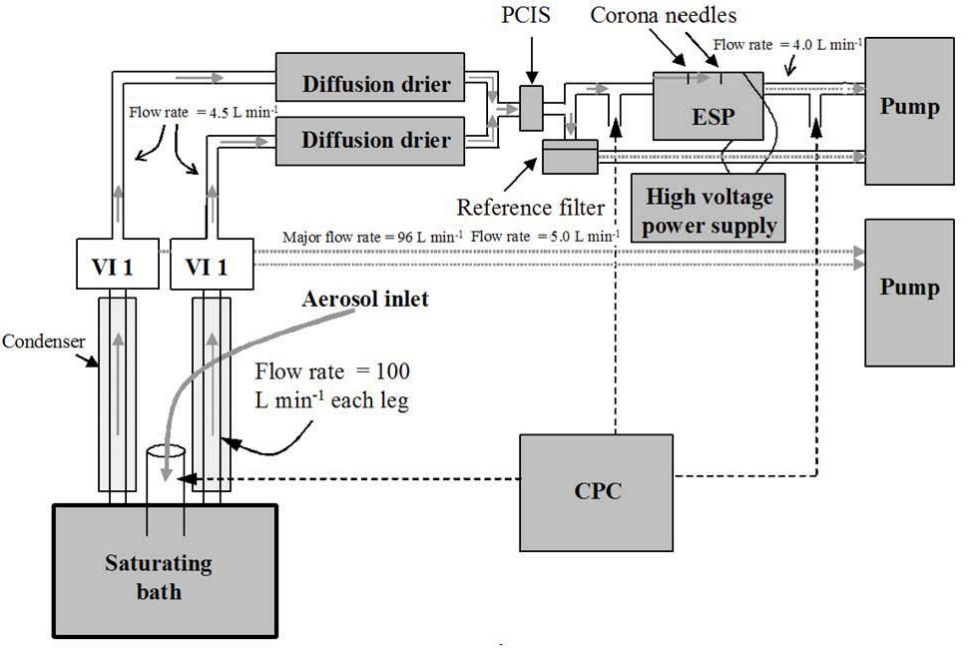
Schematic of the sampling setup used in this study for the collection of concentrated PM samples.

The in-vitro electrostatic collector has been described in detail by Sillanpää et al. [14]. The sampling system used in this field study was slightly modified from the original version and is shown in Figure 7. A Versatile Aerosol Concentration Enrichment System (VACES), operating at an intake flow of 200 l min^-1^, was used to concentrate ambient aerosols as described by Kim et al. [8,9]. Enriched aerosols passed through diffusion dryers to a Sioutas™ impactor (SKC Inc., Eighty-four, PA) [28] stage that removes particles larger than 2.5 μm in aerodynamic diameter from the air sample. When a sampling flow of 9 l min^-1 ^was drawn through this impactor, the ideal enrichment factor (EF) was 22. During some tests, an additional flow of 1.5 l min^-1 ^was added to the minor flow due to the placement of a Scanning Mobility Particle Sizer (SMPS 3936, TSI Inc.) in-line, used to monitor the system's performance, which resulted in an ideal EF of 18. The aerosols were then split into two flow streams; one (4 l min^-1^) passed through the electrostatic precipitator (ESP) where the particles were charged by corona discharge [14] and collected with a pre-cleaned Teflon-coated quartz fiber filter (Pall Gelman Sciences, 8 × 10 inches, Ann Arbor, MI) placed on the ground electrode plate; the other flow (5 l min^-1^) was used as the parallel reference sample, with concentrated PM_2.5 _samples collected on 37 mm PTFE filters (Pall Life Sciences, Teflo w/ring, PTFE membrane, porosity 2.0 μm). Parallel ambient particle samples were collected with a micro-orifice uniform deposit impactor (MOUDI; MSP Corp. Shoreview, MN) running at 30 l min^-1^. Only one impactor stage was used to remove particles larger than 2.5 μm from the sampled aerosol. The impactor stage was followed by a backup PTFE filter (Pall Life Sciences, Teflon Membrane W/PMP ring, 2.0 μm, diameter 37 mm). A condensation particle counter (CPC, Model 3022, TSI Inc., Shoreview, MN, USA) was deployed to check ambient and concentration-enriched particle number concentrations before and after ESP, in order to monitor the VACES performance and the ESP collection efficiency. The gravimetric mass concentrations from ambient MOUDI samples and the concentration-enriched reference samples were used to determine the actual enrichment factor of the VACES. Detailed particle mass and number concentration results for the various sampling sets are shown in Table 1.

During this field campaign, a voltage of 5.3 kV/cm was applied to the ESP to achieve both low particle penetration and low ozone concentrations in ESP [14]. A total of 11 sets of the filter samples were collected. Each sampling period was restricted to 2–3 hours to simulate the experimental conditions of future cell exposure studies, based on our prior work on this system demonstrating complete cell preservation and viability for sampling durations in the range of 2–3 hours [14]. In addition, shorter sampling times are expected to minimize potential ESP chemical sampling artifacts, which may be due to the reactions of corona- induced ozone and free radicals with collected PM on the ESP substrate [24,29].

In addition to the field evaluation campaign, two supplementary laboratory test series were conducted to investigate particle penetration for different particle sizes, as well as to monitor ozone generation at different applied voltages. The sampling set up for the laboratory tests was described in detail in a separate paper [14]. Ammonium sulfate particles were aerosolized with a nebulizer (VORTRAN Medical Technology Inc., Sacramento, CA, USA) and used as test aerosols. A scanning mobility particle sizer (SMPS; Model3936, TSI Inc., Shoreview, MN, USA) was operated in low flow mode (i.e., 0.3 l min^-1 ^and 3 l min^-1 ^for sample and sheath air flows, respectively) to measure the particle size distributions before and after the ESP. An ozone monitor (Model 1003-AH Dasibi Environmental Corp., Glendale, CA, USA) was used to measure the ozone production by the ESP corona electrodes at different ESP voltages.

### Gravimetric and chemical analysis

The PTFE filters of MOUDI and reference sampler were weighed with a microbalance (Model MT 5, Mettler-Toledo Inc., Highstown, NJ, USA) before and after sampling. The samples were allowed to stabilize in the weighing room for 24 hrs before weighing. A criterion for valid weighing was that duplicate mass readings were within 2 μg from each other. The relative humidity (RH) and temperature in the weighing room were 40–45% and 22–24°C, respectively. The electrostatic charges of filters were eliminated with a Po-210 radioactive source. The samples were stored in the freezer at -20 degree C and were submitted for chemical analysis at the end of the sampling campaign.

Off-line chemical analyses on the filters/substrates included ion chromatography (IC) for the analysis of five ions (chloride, nitrate, phosphate, ammonium and sulfate), selected water soluble trace elements (S, Ca, Mg, K, Fe, Zn, V, Al, Cu, Ni, Mn, Se, As and Ti) measured via inductively coupled plasma-mass spectroscopy (ICP-MS), and speciated organic compounds by gas chromatography–mass spectrometry (GC-MS) techniques. Lough et al. [30] described in detail the procedures of sample processing (e.g., filter/substrate extraction methods, digestion) for the IC and ICP-MS analyses. The extracts were analyzed by IC using a modified version of the NIOSH (National Institute for Occupational Safety and Health) Method 7903 and OSHA (Occupational Safety and Health Administration) Method 188. Speciated organic compound concentrations were measured by GC-MS techniques. Additional information on sample handling and details of the analytical procedures used in GC-MS is given by Schauer et al. [31].

## Abbreviations

CPC: Condensation particle counter; EF: Enrichment factor; ESP: Electrostatic precipitator; GCMS: Gas Chromatography Mass Spectrometry; IC: Ion chromatography; ICP-MS: Inductively coupled plasma-mass spectroscopy; MOUDI: Micro-orifice uniform deposition impactor; PAH: Polycyclic aromatic hydrocarbon; PM: Particulate matter; SMPS: Scanning mobility particle sizer; VACES: Versatile Aerosol Concentration Enrichment System.

## Competing interests

The authors declare that they have no competing interests.

## Authors' contributions

MS and PP carried out the supplementary laboratory tests. ZN and PP carried out the field sampling tests. ZN and MS analyzed the data and contributed to the writing of the manuscript. PP also participated in the writing of the manuscript. CS conceived the study, and participated in its design and coordination and helped in the writing of the manuscript. All authors read and approved the final manuscript.
